# The different outcomes in the elderly with subclinical hypothyroidism diagnosed by age-specific and non-age-specific TSH reference intervals: a prospectively observational study protocol

**DOI:** 10.3389/fendo.2023.1242110

**Published:** 2023-11-15

**Authors:** Xueqi Zhang, Yang Li, Jing Jin, Huangman Wang, Bozun Zhao, Songwen Wang, Zhongyan Shan, Weiping Teng, Xiaochun Teng

**Affiliations:** Department of Endocrinology and Metabolism, Institute of Endocrine, National Health Commission (NHC) Key Laboratory of Diagnosis and Treatment of Thyroid Diseases, The First Hospital of China Medical University, Shenyang, China

**Keywords:** subclinical hypothyroidism (SCH), TSH, natural outcomes, study protocol, reference intervals

## Abstract

**Introduction:**

Subclinical hypothyroidism (SCH) is a common endocrine disorder characterized by elevated thyroid-stimulating hormone (TSH) levels and normal free thyroxine (FT_4_) levels. The overdiagnosis and overtreatment of SCH in elderly patients have become concerns as TSH levels naturally increase with age. Studies have shown that many elderly patients with SCH can recover without treatment, and the administration of levothyroxine (L-T_4_) does not improve their prognosis. Therefore, It is necessary to establish age-specific reference ranges for TSH in elderly individuals to aid in clinical decision-making and prevent overdiagnosis.

**Methods:**

This is a multicenter prospective study that focuses on Chinese elderly patients with SCH who have TSH levels below 10 mU/L. After obtaining the informed consent of the patients, their initial diagnosis information will be registered, and they will be asked to fill out questionnaires such as the Montreal Cognitive Assessment-Basic (MoCA-B), Hamilton Depression Scale (HAMD), Hypothyroidism Symptom Questionnaire (SRQ), frail scale(FRAIL), fatigue scale, and EQ-5D. In addition, thyroid function tests, blood lipid analysis, carotid artery ultrasound, and thyroid ultrasound examinations will be conducted. Patients will also be grouped according to FT_4_ levels, the changes in FT_4_ and its relationship with TSH can also be described. For patients over 80 years old, a decrease in FT_4_ will be used as an endpoint event, while for patients between 60-80 years old, TSH levels greater than or equal to 10mIU/L or a decline in FT_4_ will be used as the endpoint event. The TSH reference intervals of the general and elderly populations will be used to calculate medical costs associated with multiple follow-ups of patients, and a social-economic analysis will also be conducted.

**Discussion:**

This study will prospectively observe elderly patients with SCH who are screened using both age-specific and non-age-specific TSH reference ranges for the elderly population. We will compare the results of elderly patients diagnosed with SCH using different reference ranges and analyze their association with FT_4_ to identify meaningful SCH patients and reduce over diagnosis and over treatment of elderly SCH.

**Ethics:**

The Medical Science Research Ethics Committee of the First Affiliated Hospital of China Medical University approved this study (ID: AF-SOP-07-1.1-01). The results will be published in an open-access journal.

**Clinical trial registration:**

https://www.chictr.org.cn/, identifier ChiCTR2300070831.

## Introduction

Subclinical hypothyroidism (SCH) is characterized by elevated serum thyroid-stimulating hormone (TSH) and normal thyroid hormone levels (1). As TSH is considered a good indicator of thyroid function, the diagnosis of SCH is primarily based on TSH levels. However, this can lead to overdiagnosis and overtreatment in elderly patients with SCH, as their TSH levels may naturally increase with age without indicating an underlying thyroid dysfunction (2).

Many studies have found that the incidence rate of SCH increases significantly with age (3, 4), which suggests that we need to consider the impact of aging on the hypothalamic-pituitary-thyroid (HPT) axis. For example, aging may weaken the circadian rhythm of TSH and reduce its responsiveness (5). According to the cross-sectional National Health and Nutrition Examination Survey (NHANES) III, the average TSH of adults aged 80 is 69% higher than that of adults aged 20 (6, 7). Data from Scottish, and Australia also confirm that TSH level increases with age (8–10). Our previous epidemiological survey in ten cities indicated that the prevalence of SCH in people over 65 years old was 19.87% according to the current TSH reference interval, but when the age-specific TSH reference range was used, the prevalence decreased to 3.3% (3). Although the mechanism is unclear, the increase in TSH in the elderly may be a normal phenomenon associated with aging. When the current diagnostic criteria are used, many elderly patients with no clinical significance will be diagnosed.

Prospective research on the natural outcome of the elderly indicated the above problem. In 2004, a study from Spain followed up 107 elderly patients with SCH over 55 years of age for 6 years (11), and the proportion of patients’ disease progression, maintenance, and recovery were 26.8%, 35.8%, and 37.4% respectively, that is, only a few patients had significant progress. In 2012, an American study conducted a 4-year follow-up of 459 elderly patients with SCH over 65 years old and found that 56% of the patients were in the state of SCH maintenance in the second and fourth years of follow-up (12). In addition, the remission rate of TPOAb-negative patients is significantly higher than that of TPOAb-positive patients. In 2017, a Chinese study conducted a 3-year follow-up of 505 patients with SCH over the age of 40 and found that only 3.4% had progressed, while 43.8% were in maintenance, and 49.7% returned to normal (13). It was also believed that TPOAb, TSH levels, creatinine, and total cholesterol levels were related to different outcomes of SCH. That is, many elderly patients with SCH may have stable or even recovery of their condition without any intervention, while only a few of them have progressed and need treatment, which suggests that using the current unified TSH reference interval of the entire population to diagnose SCH in the elderly may lead to overdiagnosis and overtreatment. To avoid this, it is recommended to establish age-specific TSH reference ranges for the elderly to better identify those who may require treatment. This approach can help prevent unnecessary interventions in patients with SCH and reduce potential adverse effects of overtreatment.

In recent years, there has been increasing attention from experts worldwide toward the management of SCH in elderly patients. Many countries have started to revise their guidelines accordingly to provide more tailored recommendations for the screening, diagnosis, and treatment of SCH in the elderly. In 2020, the French expert consensus proposed that individualized treatment should be adopted for elderly patients with SCH (14). In 2021, experts from China also advocated jointly determining whether treatment is needed according to age, TSH level, symptoms, cardiovascular risk factors, etc (15). The guidelines of other regions are listed in **Table 1**. These guidelines have great differences and lack reliable epidemiological research support, which is also one of the purposes of our prospective research.

**Table 1:** Current guidance on the treatment of subclinical hypothyroidism in the elderly.

No.	Publication year	Organization	Main recommendations	
			TSH<10mIU/L	TSH≥10mIU/L
1 (16)	2012	American Thyroid Association (ATA)	Consider treatment if there are symptoms of hypothyroidism, TPOAb(+) or atherosclerosis, cardiovascular disease, heart failure, or risk factors of these diseases	Consider treatment
2 (17)	2013	European Thyroid Association (ETA)	Age<70 years: treat if there are symptoms; observe if there is no symptom	Age<70 years: treat
			Age>70 years: observe	Age>70 years: consider treatment if there are symptoms or cardiovascular risk factors
3 (18)	2013	The Thyroid Department of the Brazilian Society of Endocrinology and Metabolism	Age ≤ 65 years: observe if there are no comorbidities; consider to treat if it is possible to progress to hypothyroidism; if there is cardiovascular disease or its risk, consider to treat if TSH≥7 mIU/L; if there are hypothyroidism symptoms, the therapeutic test should be considered	Treat
			Age>65 years: observe	Treat
4 (19)	2018	National Institute for Health and Care Excellence (NICE)	Age <65 years: consider trial	Age <70 years: treat
			Age≥65 years: watch and wait	Age≥70 years: watch and wait
5 (20)	2020	Gulf Cooperation Council (GCC) Countries	TPOAb(+): treat	Treat
			TPOAb(-): treat if there are indications; otherwise, observe	
6 (15)	2021	Endocrine Metabolic Diseases Group of the Chinese Geriatrics Society, Thyroid Group of the Chinese Society of Endocrinology, Chinese Medical Association	60-70 years old: treat if TPOAb(+) or there are hypothyroidism symptoms or cardiovascular risk factors; otherwise, observe	60-70 years old: treat
			71-80 years old: observe	71-80 years old: treat if there are hypothyroidism symptoms or cardiovascular risk factors; otherwise, observe
			Age>80 years: obersve	Age>80 years: obersve

In the process of SCH, we need to consider the adverse outcomes of cardiovascular and cognitive functions. The thyroid hormone is closely related to the cardiovascular system and plays a key role in the regulation of cardiac muscle cell inotropic and chronotropic effects (21, 22). SCH is generally considered a risk factor for cardiovascular disease and cardiovascular-related death events. At present, some studies have shown that the prediction of cardiovascular disease and mortality in elderly patients with SCH should be based on age and TSH level (23–26). The prognosis of the elderly is good, and TSH>10mU/L is often associated with the occurrence of cardiovascular diseases, which need further confirmation. For cognitive function, the thyroid hormone plays a key role in brain development and maturation, so abnormal thyroid function and hormone secretion may affect the neuropsychiatric system (27). Although thyroid function examination is usually performed in patients with cognitive impairment or depression, the relationship between SCH and neurological and psychological functions is still unclear. However, previous research suggested that SCH in the elderly has nothing to do with cognitive decline or dementia events, so it may not be necessary to conduct SCH screening in the elderly with cognitive decline (28–30). It is noteworthy that some studies have found that elderly patients with SCH may be prone to depression, but the results are inconsistent (31–33). Therefore, our research will focus on the cardiovascular disease and depression of patients.

## Methods

### Research type

This study adopts the methodological strategy of the cohort study in analytical epidemiology to carry out various data collection, diagnostic test analysis around the gold standard and the test data of diagnostic subjects, and economic analysis.

### Research objectives and main results

The goal of this study is to prospectively follow up on elderly patients with SCH who are diagnosed using age-specific TSH reference intervals and non-age-specific TSH reference intervals, which aims to establish TSH reference intervals for different age groups to improve the diagnosis and management of SCH in the elderly. The main outcomes being assessed include thyroid function, hypothyroidism-related symptoms, cardiovascular events, cognition, depression, and fatigue, as well as economic and social benefits analysis. The above information will help make informed decisions about the best course of treatment for elderly patients with SCH.

### Study population

We are conducting a prospectively observational study on patients over 60 years of age diagnosed with SCH. The study was conducted in the First Affiliated Hospital of China Medical University and the West China Hospital of Sichuan University. Patients will be included from December 2022 to December 2023, and the follow-up data will be collected until December 2025.

### Inclusion criteria and exclusion criteria

Inclusion criteria:

Elderly patients with SCH will be included according to the elderly and the general population-specific reference ranges, respectively.

(1) Inpatients and/or outpatients aged≥60 years.
(2) The levels of free triiodothyronine(FT_3_) and FT_4_ are within the normal range.
(3) TSH is greater than the upper limit of the normal reference range (old standard) and less than the upper limit of the normal reference range (new standard).

Exclusion criteria:

(1) Does not meet the inclusion criteria, does not sign the consent form, or participates involuntarily.
(2) Exit halfway due to various reasons.
(3) Unable to cooperate with the project team to complete all inspection contents.
(4) Other factors leading to TSH increase. 1) increased TSH caused by measurement errors; 2) convalescence stage of the euthyroid sick syndrome; 3) central hypothyroidism; 4) renal insufficiency; 5) lack of glucocorticoids; 6) the recovery stage of thyroid function in subacute thyroiditis; 7) having taken amiodarone or lithium; 8) other interfering factors.

### Public involvement

All patients will participate in the study voluntarily and sign an informed consent form. This study will be based on patient and public participation. We will complete the questionnaire by asking patients questions and promoting medical knowledge by establishing good relationships with patients.

### Study follow-up assessments

**Figure 1:**
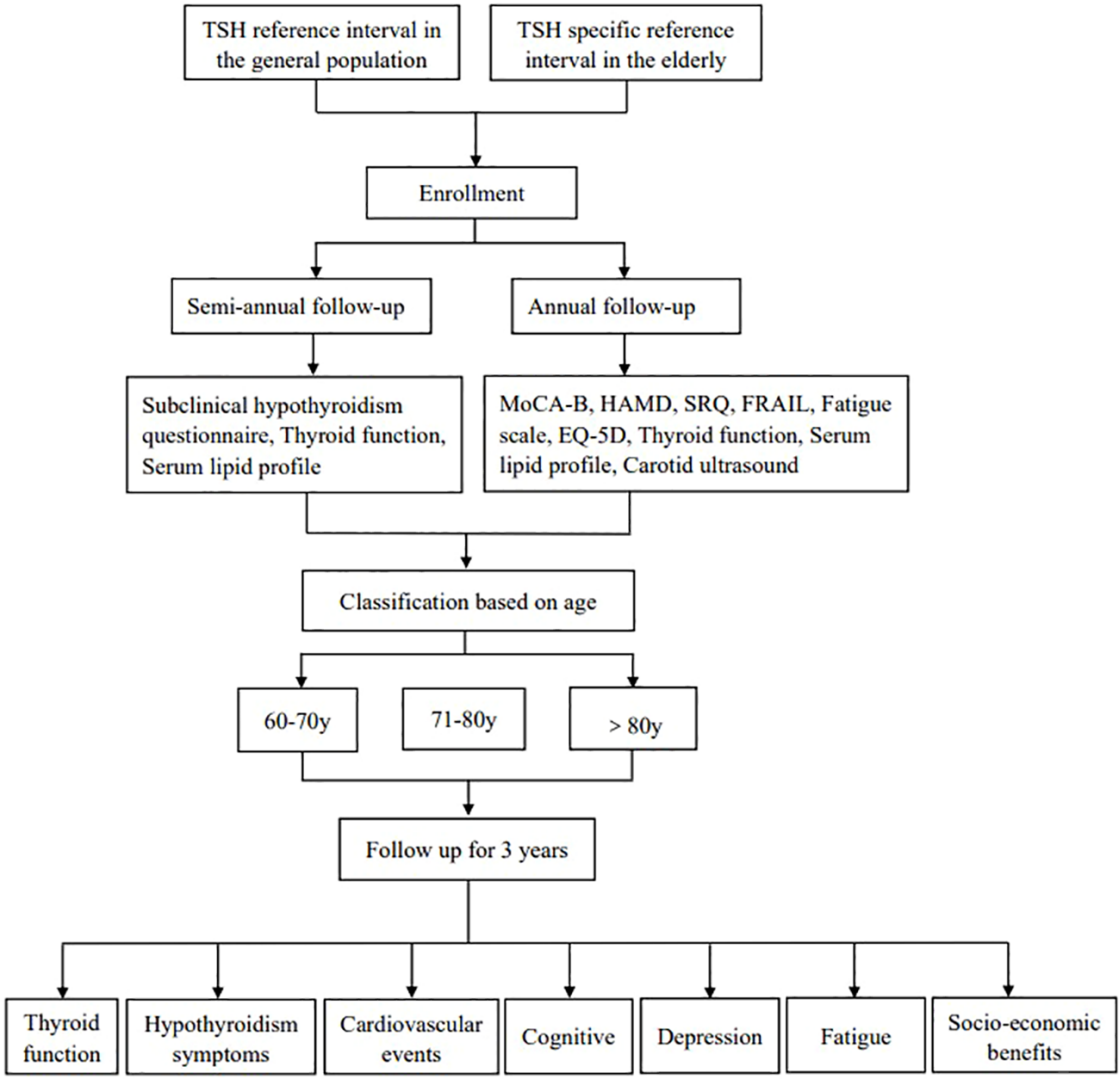
Study process.

When a patient has FT_4_ levels below the lower limit of the normal reference range during follow-up, follow-up is terminated and thyroid hormone replacement therapy will be initiated. For patients aged 60 to 70 years, start treatment when TSH≥10mIU/L. For patients aged 71 to 80 years, treatment begins when TSH≥10 mIU/L and is accompanied by symptoms of hypothyroidism. Normal value reference ranges will be established based on the TSH levels of 120 normal elderly individuals over 60. Elderly SCH patients screened according to the TSH reference interval in the normal population and the elderly population will be followed up every 6 months, including the subclinical hypothyroidism questionnaire, the Montreal Cognitive Assessment-Basic (MoCA-B), Hamilton Depression Scale (HAMD), Hypothyroidism Symptom Questionnaire (SRQ), frail scale(FRAIL), fatigue scale, EQ-5D, thyroid function, blood lipid analysis, carotid color ultrasound, and thyroid ultrasound. The estimated sample size is 438. Patients diagnosed with different TSH reference intervals will be compared for cognitive function, depression scores, hypothyroidism, fatigue severity, carotid atherosclerosis, lipid profile, and cardiocerebrovascular complications. Besides, TSH reference intervals will be applied to calculate medical costs and evaluate the economic benefits of multiple follow-ups of patients with SCH. The research design can be seen in **Figure 1**.

The scoring breakdown of MoCA-B is as follows: visuospatial and executive functioning: 5 points; animal naming: 3 points; attention: 6 points; language: 3 points; abstraction: 2 points; delayed recall (short-term memory): 5 points; orientation: 6 points; education level: 1 point is added to the score if they have 12 years or less of formal education. A total score of 26 or greater is within the normal range. Using HAMD to assess the degree of depression: 10-13 mild; 14-17 mild to moderate; >17 moderate to severe. The FRAIL consists of five questions with a score of 0-5 (0: for the best state, 5: for the worst state), 3-5 for frailty, 1-2 for pre-frailty, and 0 for physical fitness.

### Research quality control

The collection of questionnaire information and the biological specimen will be conducted by doctors, nurses, or investigators with work experience. Data management and quality control will be carried out for data entry, export, storage, and other aspects. Concerning the quality control of biological samples, the collection, pretreatment, transfer, freezing, and other related processes will be carried out in strict accordance with the relevant Standard Operation Procedure standards. At the same time, random inspection will be carried out on all kinds of work from sample collection to detection to ensure sample quality.

The testing and laboratory processing units are the Laboratory of the First Affiliated Hospital of China Medical University and the West China Hospital, Sichuan University. This study will strictly follow the hospital’s laboratory processing approval process for filing and operation.

Concerning data management and security, all kinds of data in this study will be uniformly saved, and all members, temporary employees, and students will sign a confidentiality agreement.

### Data analysis

We will calculate age-specific TSH reference intervals based on the obtained data, analyze the natural outcomes, the occurrence of cardiovascular events, cognitive status, depression, and fatigue of elderly patients with SCH diagnosed under different reference intervals, and conduct economic analysis.

Continuous data will be expressed as mean ± standard deviation or median with a range of quartiles. Classification data will be expressed in numbers with percentages. Cox proportional risk model will be used to evaluate survival results. The variables measured several times during the follow-up period will be compared with the paired t-test according to the clinical management used. The significance level will be set to 0.05, and R software, prodlim, and Cairo software will be used to analyze the data.

### Limitations

Due to the significant variability in TSH levels, we cannot guarantee whether the diagnosis of patients has been affected by the fluctuations in TSH. We can only strive to have patients undergo two repeated measurements whenever possible, and make an effort to select the same time point for testing. Considering the significance of T_4_, we plan to establish a diagnostic indicator that combines both T4 and TSH.

## Discussion

The specific TSH reference interval of elderly patients with SCH and its management deserve attention (34). In recent years, experts have recognized that the management of elderly patients with SCH needs to follow the principle of individualization (35), and much current research indicated that L-T_4_ has not shown any improvement in the treatment of cardiovascular disease mortality, cognitive function, depression, fatigue, hypothyroidism and other symptoms of elderly patients (36–40), which make us consider that the reference range of TSH for the elderly needs to be revised. Distinguishing from the reference interval of TSH for the whole population will effectively identify the truly meaningful patients with SCH, avoid over-diagnosis and save social resources.

To confirm its necessity and establish an age-specific TSH reference interval, we conducted this prospective study of the natural outcomes of elderly patients with SCH diagnosed by different reference ranges of TSH. In addition, the economic benefits will also be evaluated. In a word, our study will facilitate the accurate discrimination of high-risk patients in future clinical diagnosis and treatment, and avoid overdiagnosis and overtreatment.

## Data availability statement

The original contributions presented in the study are included in the article/supplementary material. Further inquiries can be directed to the corresponding author.

## Author contributions

XZ is the first author of this study. XT is the corresponding author supervising this work. XZ drafted the manuscript. YL provided major technical support. JJ, HW, BZ and SW assisted in the literature review. ZS and WT checked the manuscript. All authors contributed to the article and approved the submitted version.
